# Next-Generation Sequencing Reveals the Role of Epigallocatechin-3-Gallate in Regulating Putative Novel and Known microRNAs Which Target the MAPK Pathway in Non-Small-Cell Lung Cancer A549 Cells

**DOI:** 10.3390/molecules24020368

**Published:** 2019-01-21

**Authors:** Vaishali Bhardwaj, Abul Kalam Azad Mandal

**Affiliations:** Department of Biotechnology, School of Bio Sciences and Technology, Vellore Institute of Technology, Tamil Nadu, Vellore 632014, India; shaali235@gmail.com

**Keywords:** next-generation sequencing, microRNAs, A549, EGCG, MAPK pathway

## Abstract

Lung cancer constitutes 85% of non-small cell lung cancer diagnosed cases. MicroRNAs are novel biomarkers that are capable of modulating multiple oncogenic pathways. Epigallocatechin-3-gallate (EGCG) is a potent chemopreventive and chemotherapeutic agent for cancer. We aimed to identify important known and putative novel microRNAs modulated by EGCG in A549 cells using next-generation sequencing and identify their gene targets. Preliminary analysis revealed an IC50 value of 309 μM with G0/G1 phase arrest at 40 μM EGCG treatment. MicroRNA profiling identified 115 known and 4 putative novel microRNAs in 40 μM and 134 known and 3 putative novel microRNAs in 100 μM EGCG-treated A549 cells. The top 10 up-expressed microRNAs were similar between the untreated control and EGCG-treated A549 cells. An up-expression in oncogenic microRNAs, which belong to broadly conserved seed families, were observed in untreated control and EGCG-treated A549 cells. Kyoto Encyclopedia of Genes and Genomes and Protein Analysis Through Evolutionary Relationships pathway analyses of the validated microRNA targeting genes strengthened the hypothesis that EGCG treatment can modulate microRNAs that play a significant role in the MAPK signaling pathway. Expression profile of microRNAs was validation by quantitative real time PCR of randomly selected microRNAs. This study identified signature microRNAs that can be used as novel biomarkers for lung cancer diagnosis.

## 1. Introduction

Green tea, brewed from the unfermented dry leaves of the plant *Camellia sinensis*, is the most consumed non-alcoholic beverage in Asian countries and is now gaining popularity in western countries as well. It contains a wide range of phytochemicals which exhibits anti-cancer, anti-oxidative, and anti-inflammatory properties [1]. Among these phytochemicals, (−)-epigallocatechin-3-gallate (EGCG) accounts for 18–36% of the total phenolic compounds and 70% of the catechins present in green tea. Animal and cell line studies have demonstrated an important role of EGCG in promoting apoptosis and reducing cancer growth. EGCG is a potential chemopreventive and chemotherapeutic compound against skin [2], lung [3], breast [4], colon [5], prostate [6], and other cancers [7,8]. 

Lung cancer dominates among all the cancers with the leading mortality rate worldwide, 85% of which are non-small cell lung cancer (NSCLCs) cases [9]. Over 50% of the lung cancer patients die within a year of diagnosis with an estimated 1.6 million deaths per year [10]. The prevalent histological subtypes of NSCLCs include lung adenocarcinoma (LUAD), lung squamous cell carcinoma (LUSC) [11], and large cell lung cancer, out of which LUSC and large cell lung cancer are associated with smoking [12]. Clinical studies have showed that interaction between chemotherapeutic drugs and nicotine present in cigarette reduce the efficiency of chemotherapy in lung cancer patients by stimulating the cell survival pathways [13]. 

Despite many improvements in the field of early cancer diagnosis, the frequency of advanced stage detection is still notable. Tyrosine kinase inhibitors (TKIs) provide temporary benefits towards NSCLC resistance. However, EGFR (Epidermal growth factor receptor) mutations and ALK (Anaplastic lymphoma kinase) translocation are major challenges for TKIs in clinical practices [14]. Liquid biopsy study after drug therapy, radiological assessment as well as after and post-surgical adjuvant therapy explored circulating tumor cells (CTCs) and circulating tumor DNA (ctDNA) as useful biomarkers. These biomarkers are very useful to monitor TKIs which play an important role in detecting drug mutations [15]. Resistance development in cancer cells is the main challenge towards targeted therapy. The consensus analysis of liquid biopsies revealed CTCs and ctDNA/miRNA as important biomarkers for tumor profiling [15]. Thus, identification of novel diagnostic biomarkers and treatment approaches are the prerequisite for designing an optimal therapeutic regime. Recently, the cancer researchers have drifted their focus on the importance of microRNAs controlling expression of the target mRNAs which facilitates angiogenesis, tumor invasion, and growth [16,17].

Signaling pathways namely the RAS/RAF/MAPK [18], PI3K/AKT [19,20], JAK/STAT [21] and wnt/β-Catenin [22] are very crucial for cancer growth and proliferation. The MAPK cascade plays an important role in human cancer cell survival, proliferation, and drug resistance [23]. It is an evolutionarily conserved, developmental pathway, which regulates tissue homeostasis and organ development. A transmitted signal cascade from receptor tyrosine kinases (RTKs), RAS and RAK family is relayed to important signaling molecules namely mitogen-activated extracellular signal-regulated kinase 1/2 (MAP2K1/2), and extracellular signal-regulated kinase 1/2 (MAPK3/1). Activated ERK1/2 nuclear translocation triggers transcriptional activation of many oncogenes, which modulates cellular processes responsible for cell proliferation, migration, angiogenesis, and cell survival [24]. Previous studies have identified a significant role of microRNAs in regulating MAPK signaling cascade in lung cancer [25,26]. The high prevalence rate of MAPK pathway in cancer regulation has motivated the oncology researchers in targeting critical pathway nodes of microRNAs and MAPK pathway.

MicroRNAs are a novel class of short, single-stranded, evolutionarily conserved non-coding RNA molecules (19–22 nucleotides in length), which play an important role in post-transcriptional gene regulation and modulation of biological processes including cell differentiation, proliferation, apoptosis, maintenance of cellular homeostasis etc. [27]. Extensive research on transcription regulation by microRNAs including oncogenes, proto-oncogenes and tumor suppressor genes [28,29,30] has explored the fundamental principles in the field of ncRNAs. Genome-scale mapping for microRNA profiling is a high throughput method for the identification of known and prediction of novel microRNAs [31]. MicroRNA profiling has validated that the altered expression of these microRNAs describes several pathologies including cancer [32,33,34,35] and these microRNAs can serve as a useful diagnostic and prognostic biomarker in lung cancer. The role of various microRNAs has been widely identified in lung cancer in which MAPK signaling cascade is known to play a significant role.

In the present study, we aimed to analyze the effect of EGCG on known as well as putative novel microRNAs in A549 cells and identify their gene targets for understanding their role in cancer pathways. Furthermore, qRT-PCR validation of selected microRNAs was performed to endorse our next-generation sequencing (NGS) data.

## 2. Results

### 2.1. EGCG Induced G0/G1 Phase Arrest in A549 Cell Line

**Figure 1:**
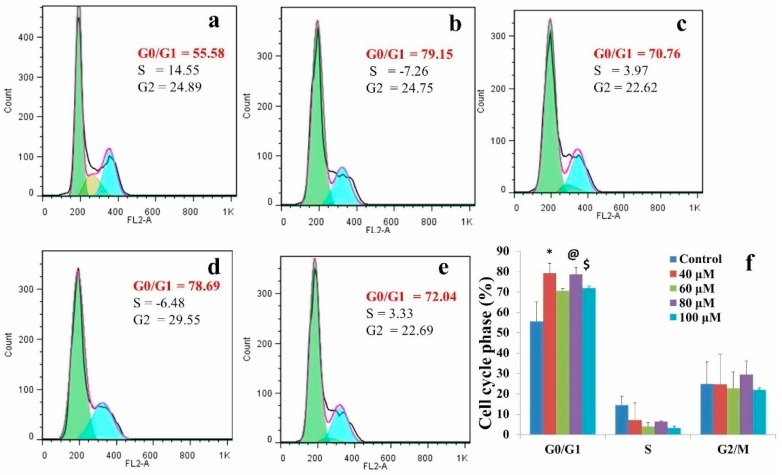
EGCG induced arrest at the G0/G1 phase of the cell cycle in A549 cells. (**a**) Untreated control (**b**) 40 µM EGCG treatment (**c**) 60 µM EGCG treatment (**d**) 80 µM EGCG treatment and (**e**) 100 µM EGCG treatment (**f**) the cell cycle distribution analysis. The data are shown as mean ± SD. Significant (*p* < 0.05) difference between the groups are indicated by: “*” between untreated control and 40 µM EGCG treatment, “@” between untreated control and 80 µM EGCG treatment, and “$” between untreated control and 100 µM EGCG treatment.

EGCG treatments significantly increased the percentage of cells at the G0/G1 phase of the cell cycle (Figure 1). The percentage of cells at the G0/G1 phase of the cell cycle increased from untreated control (55.58%) to 40 μM EGCG treatment (79.15%). Furthermore, we observed that 79.15, 70.76, 78.76, and 72.04% of cells were persistent at the G0/G1 phase of the cell cycle at 40, 60, 80, and 100 μM EGCG treatments, respectively. A rational cell percentage at the G0/G1 phase of the cell cycle beyond 40 μM EGCG treatment attests to G0/G1 phase arrest at 40 μM EGCG treatment (Figure 1). In addition, no apoptosis was observed in EGCG-treated A549 cells. 

### 2.2. Analysis of MicroRNAs

**Figure 2:**
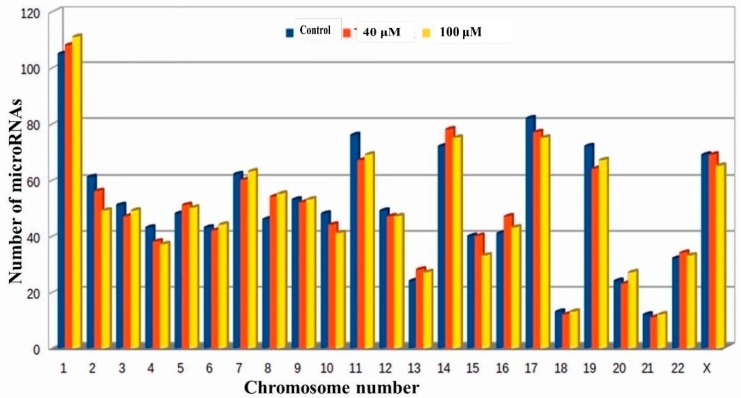
Chromosomal distribution of known microRNAs in the untreated control, 40 and 100 μM EGCG treatments. Color key- blue: untreated control A549 cells, red: 40 μM EGCG treatment and yellow: 100 μM EGCG treatment.

The miRBase-21 database was used for known miRNA detection using sequence similarity approach (ncbi-blast-2.2.30). Novel microRNA sequences were predicted using Mireap_0.22b [36]. In total, 958, 944 and 935 known microRNAs were detected in the untreated control, 40, and 100 μM EGCG treatments, respectively. MicroRNAs with ≥50 read count was counted as 206, 194, and 199 for untreated control, 40, and 100 μM EGCG treatments, respectively. In addition, on an average, maximum microRNAs were predicted from chromosome 1 followed by chromosome 17 and 14 (Figure 2). About 105, 108, and 111 microRNAs were predicted from chromosome 1 followed by 82, 77, and 75 from chromosome 17 in the untreated control, 40, and 100 μM EGCG treatments, respectively.

### 2.3. A549 Cell Associated MicroRNA Expression Signature

**Figure 3:**
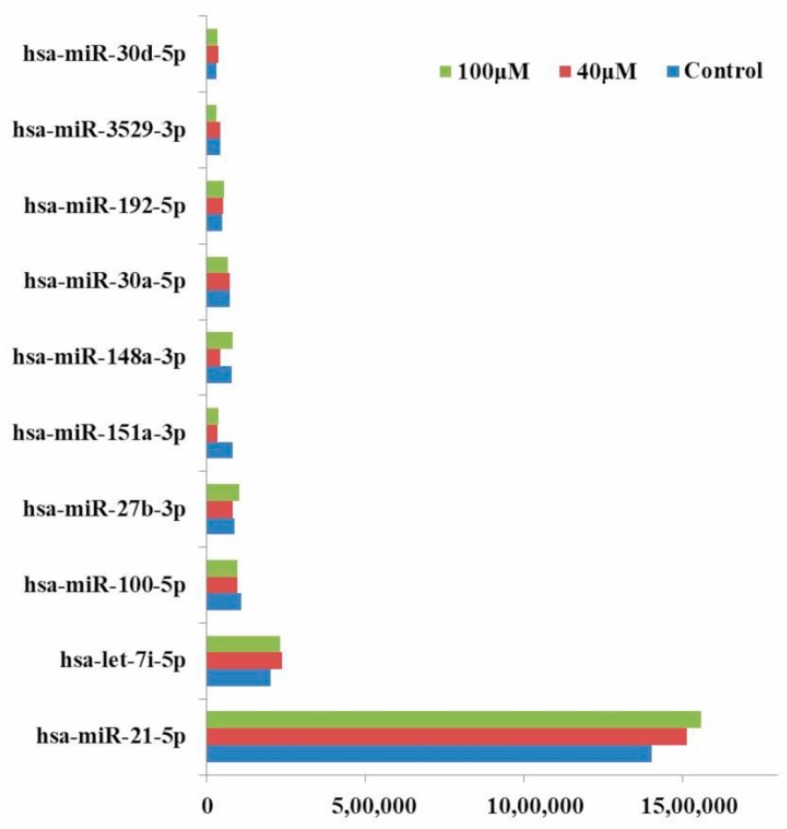
Read count of top ten microRNAs expressed in A549 cells after 40 and 100 μM EGCG treatments.

We identified top ten up-expressed microRNAs (hsa-miR-21-5p, hsa-let-7i-5p, hsa-miR-100-5p, hsa-miR-27b-3p, hsa-miR-151a-3p, hsa-miR-148a-3p, hsa-miR-30a-5p, hsa-miR-192-5p, hsa-miR-3529-3p, and hsa-miR-30d-5p) in untreated control, 40 and 100 μM EGCG treatments by the integrated analysis (Figure 3). Hsa-miR-21-5p was significantly up-expressed in the untreated control, 40, and 100 μM EGCG treatments with the read count of 1403229, 1511476, and 1557436 respectively, followed by hsa-miR-7i-5p with 202380, 238919, and 231472 read counts in the untreated control, 40, and 100 μM EGCG treatments, respectively. Surprisingly, these ten up-expressed microRNAs showed consistent up-regulation in the untreated control vs. 40 and the untreated control vs. 100 μM EGCG treatments (Figure 3). The microRNA sequencing data indicated that top ten up-expressed microRNAs expression was not affected by EGCG treatment. Majority of these signature microRNAs belonged to the broadly conserved seed families namely let-7, hsa-miR-21, and hsa-miR-30. 

### 2.4. Prediction of Putative Novel MicroRNAs

**Figure 4:**
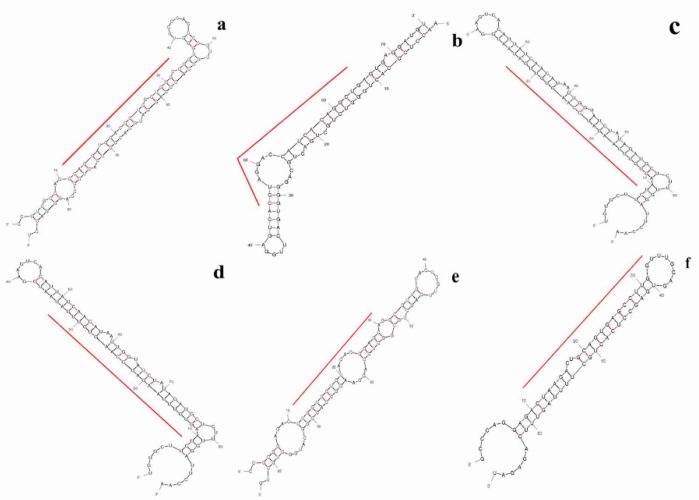
Predicted secondary structures of selected putative pri-miRNA sequences in the untreated control, 40, and 100 μM EGCG treatments. The red line signifies the location of the putative mature microRNA sequence in the secondary structure. (**a**) CCAGGAUGCACGCUCGCUGGGCU; (**b**) CCUAGGACCAUCACAGGCUGA; (**c**) CAGAUGGUUGCUGAUCUGUGCA; (**d**) AGUGUUUAAGAUCCAAGUGUUG; (**e**) UUGGUGGUGUACACGGAGCAG; (**f**) CUAAGACUGCAGUGAGCCUUGGU.

We identified top ten up-expressed predicted putative novel microRNA sequences in control, 40, and 100 μM EGCG treatments and named as TPP-A549-1, TPP-A549-2 and so on. The chromosomal location, precursor, and mature sequences of the predicted putative novel miRNAs are presented in Table 1. We observed six putative novel microRNA sequences which were expressed in more than one sample. The predicted secondary structure of these putative novel microRNAs is presented in Figure 4. The putative novel microRNA TPP-A549-7 was expressed in all the samples with the MFE (Minimum free energy) value of -29 Kcal/mol (Table 1). In the untreated control, the putative novel microRNA TPP-A549-1 was highly expressed with the read count of 3852. In addition, the putative novel microRNAs TPP-A549-11 and TPP-A549-17 were up-expressed in 40 and 100 μM EGCG treatments with the read count of 998 and 1334 respectively (Table 1). 

### 2.5. Differential Expression Analysis of Known MicroRNAs

**Figure 5:**
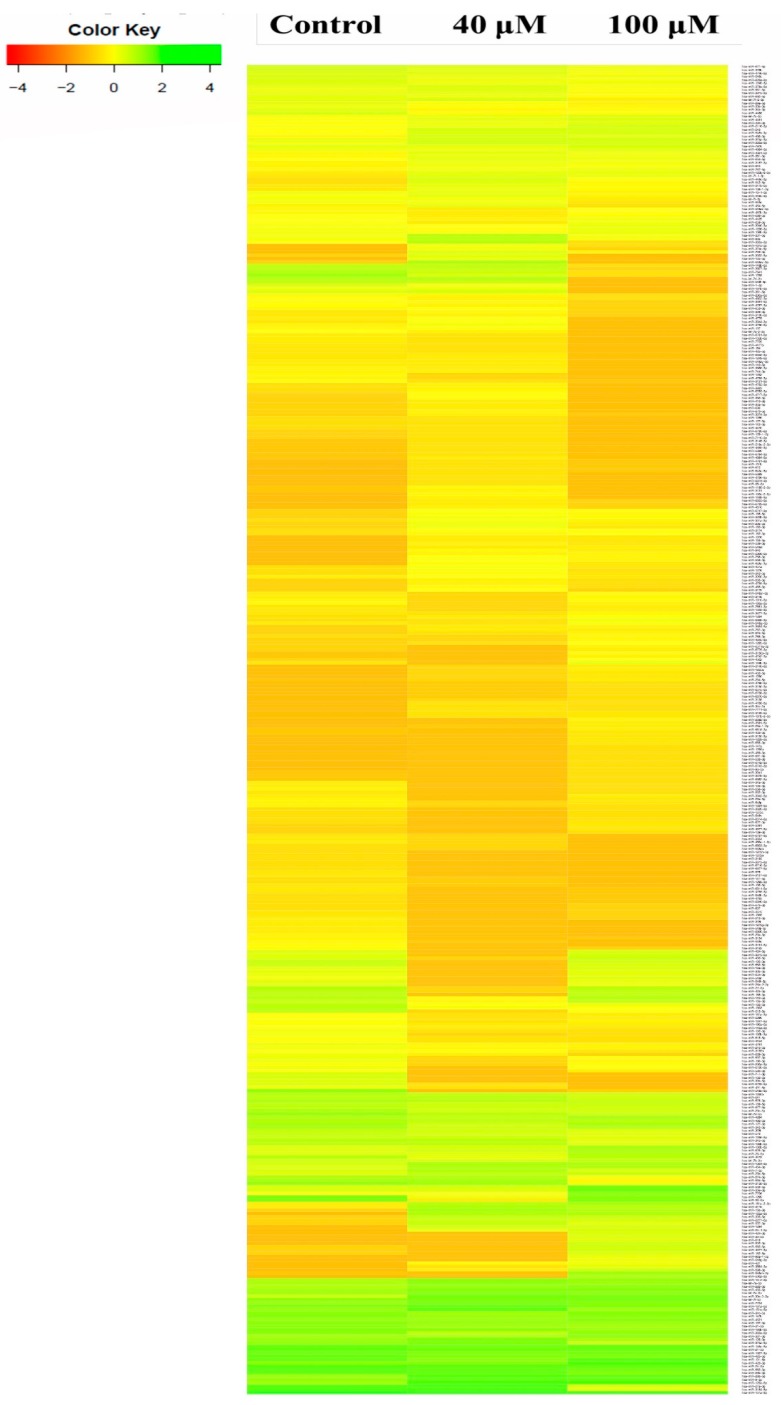
The expression profiles of known microRNAs in the untreated control, 40 μM, and 100 μM EGCG treatments. Color key- red: up-regulation, green: down-regulation and yellow: neutral expression.

**Figure 6:**
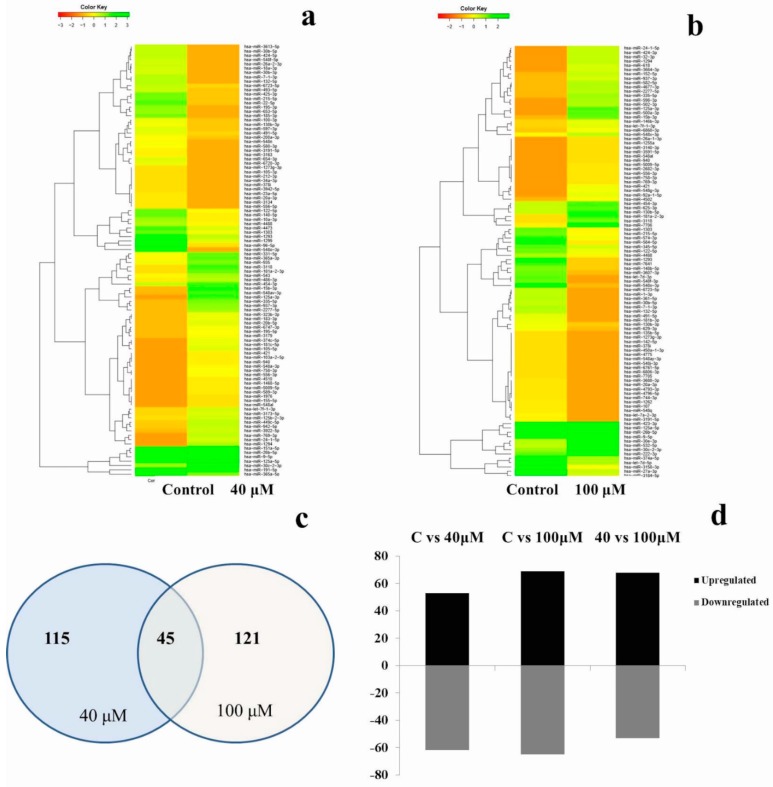
Dose-dependent microRNA expression profiles of top 100 microRNAs. (**a**) Relative microRNA expression levels in untreated control vs. 40 μM EGCG treatment; (**b**) Relative microRNA expression levels in untreated control vs. 100 μM EGCG treatment; (**c**) Venn diagram depicting the dose-dependent responses of microRNAs to EGCG; (**d**) Summary set of up- and down-regulated microRNAs exhibiting greater than 2 log2-fold change expression after EGCG treatments. Color key- red: up-regulation, green: down-regulation and yellow: neutral expression.

A complete microRNA profiling is depicted in Figure 5, indicating the effect of EGCG on A549 cells. MicroRNA expression with greater than 2 log2 fold change was determined in the untreated control vs. 40 μM and the untreated control vs. 100 μM EGCG treatments. The heat maps of the top 100 differentially expressed microRNAs between the samples are presented in Figure 6a,b. 

A complete list of up- and down-regulated microRNAs is presented in Table 2. We found 115 microRNAs differentially expressed in the untreated control vs. 40 μM EGCG treatment, and 134 microRNAs differentially expressed in control vs. 100 μM EGCG treatment (Figure 6c). Out of the 115 differentially expressed microRNAs reported in the untreated control vs. 40 μM EGCG treatment, 53 were up- and 62 were down-regulated. Furthermore, in the untreated control vs. 100 μM EGCG treatment, we reported 69 up- and 65 down-regulated microRNAs (Figure 6d). 

**Figure 7:**
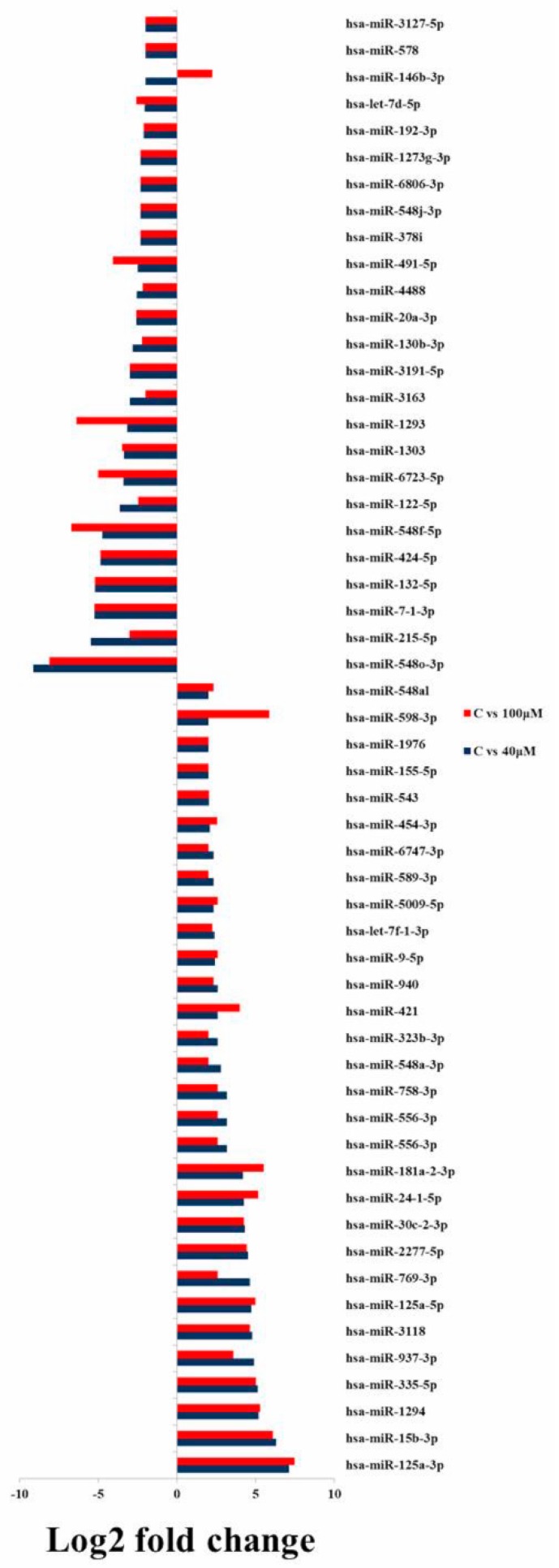
Common up- and down-regulated microRNAs between untreated control vs. 40 and the untreated control vs. 100 μM EGCG treatments.

By comparing the data with all the reported up-regulated microRNAs, hsa-miR-125a-3p showed the highest change of log2 fold expression in the untreated control vs. 40 μM EGCG treatment (7.12 log2 fold change) and untreated control vs. 100 μM EGCG treatment (7.47 log2 fold change). Furthermore, hsa-miR-548o-3p was down-regulated by -9.12 and -8.12 log2 fold change in the untreated control vs. 40 μM EGCG and the untreated control vs. 100 μM EGCG treatments, respectively. We observed 21 up- and 24 down-regulated microRNAs in the untreated control vs. 40 and the untreated control vs 100 µM EGCG treatments (Figure 7). 

### 2.6. Differential Expression Analysis of Putative Novel MicroRNA Sequences 

**Figure 8:**
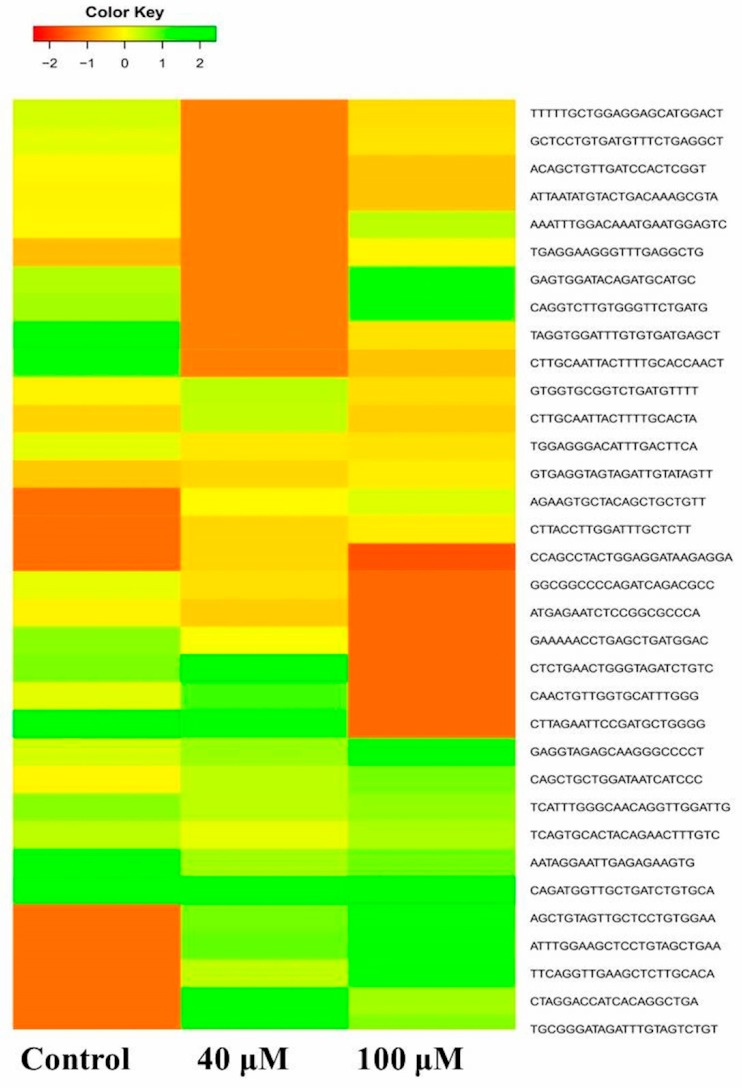
The expression profiles of putative novel microRNAs in the untreated control, 40, and 100 μM EGCG treatments. Color key- red: up-regulation, green: down-regulation and yellow: neutral expression.

**Figure 9:**
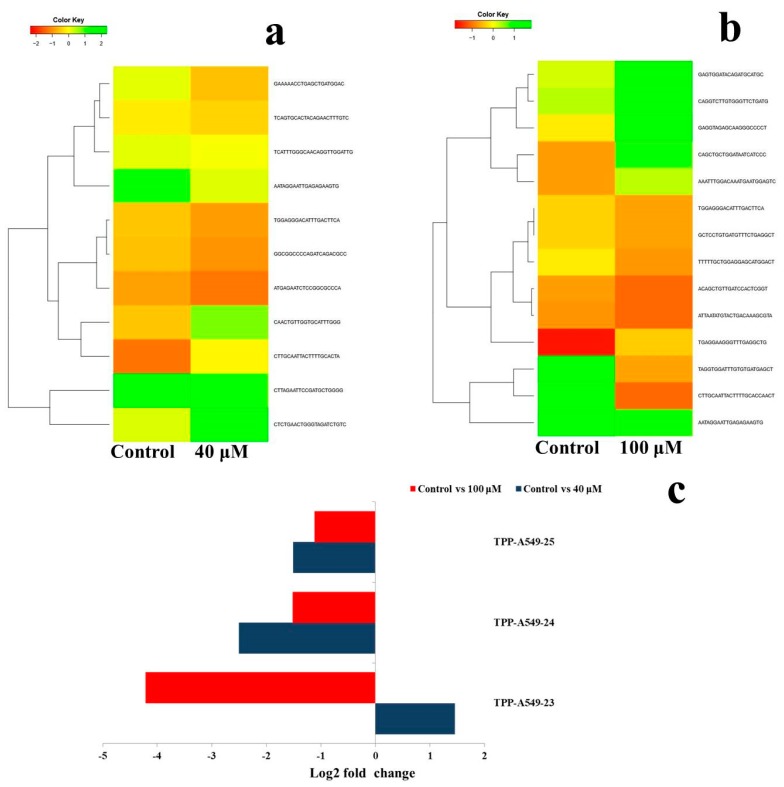
The differential expression pattern of putative novel microRNAs. (**a**) Untreated control vs. 40 μM EGCG treatment; (**b**) Untreated control vs. 100 μM EGCG treatments; (**c**) Log2 fold change of three putative novel microRNAs which were common in untreated control vs. 40 and untreated control vs. 100 μM EGCG treatment. Color key- red: up-regulation, green: down-regulation and yellow: neutral expression.

Heat maps were plotted to study the differential expression pattern of putative novel microRNAs. A complete putative microRNA profiling is shown in Figure 8. Significantly differentially expressed putative novel microRNAs are plotted as heat maps as shown in Figure 9a,b. Comparison of differential expression of putative novel microRNAs in the untreated control vs. 40, the untreated control vs. 100, and 40 vs. 100 μM EGCG treatments showing greater than 2 log2 fold change revealed 4 putative novel microRNAs differentially expressed in control vs. 40, 3 in control vs. 100, and 4 in 40 vs. 100 μM EGCG treatment. In the untreated control vs. 40 μM EGCG treatment, the putative novel microRNA TPP-A549-26 was up-regulated and three others namely TPP-A549-24, TPP-A549-27, and TPP-A549-28 were down-regulated. In addition, in the untreated control vs. 100 μM EGCG treatment, putative novel microRNAs TPP-A549-32 and TPP-A549-29 were up-regulated and TPP-A549-30 and TPP-A549-31 were down-regulated (Table 3). The chromosomal location, precursor, and mature sequence details of greater than 2 log2 fold change of putative novel microRNAs in the untreated control vs. 40 and the untreated control vs. 100 μM EGCG treatments are presented in Table 3. Three putative novel microRNAs namely TPP-A549-23, TPP-A549-24, and TPP-A549-25 were commonly differentially expressed in the untreated control vs. 40 and the untreated control vs. 100 μM EGCG treatments (Figure 9). It was interesting to observe that putative novel microRNA TPP-A549-23 was up-regulated in the untreated control vs. 40 μM EGCG treatment and was down-regulated in the untreated control vs. 100 μM EGCG treatment (Figure 9c). 

### 2.7. qRT-PCR Analysis of MicroRNAs

**Figure 10:**
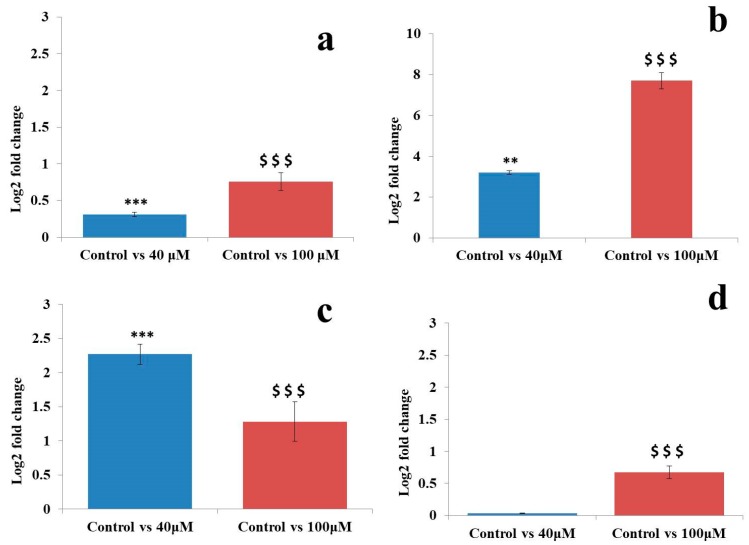
Validation of randomly selected known microRNAs by qRT-PCR. (**a**) Log2 fold change of miR-21-5p; (**b**) Log2 fold change of hsa-miR-548o-5p; (**c**) Log2 fold change of hsa-miR-181c; (**d**) Log2 fold change of hsa-miR-212-5p microRNAs. The significant difference between the untreated control vs. 40 and the untreated control vs. 100 μM EGCG treatmts are indicated by ‘*’ between untreated control and 40 µM EGCG treatment, and ‘$’ between untreated control and 100 µM EGCG treatment. Significance levels of *p* < 0.001 (‘***’; ‘$$$’), *p* < 0.01 (‘**’) are denoted by repetition of these symbols.

The qRT-PCR analysis was performed to validate the NGS dataset. Hsa-miR-21-5p, hsa-miR-548o-5p, hsa-miR-181c, and hsa-miR-212-5p microRNAs were randomly selected for the study. qRT-PCR analysis showed 3.2, and 2.17 log2 fold change in expression in the untreated control vs. 40 μM EGCG treatment and 2.5 and 1.28 log2 fold change in expression in the untreated control vs. 100 μM EGCG treatment in hsa-miR-548o-5p and hsa-miR-181c, respectively. Furthermore, minimal differential log2 fold change expression of 0.31 and 0.03 in the untreated control vs. 40 μM EGCG treatment and 0.76 and 0.6 in the untreated control vs. 100 μM EGCG treatment was observed in hsa-miR-21-5p and hsa-miR-212-5p respectively (Figure 10).

**Figure 11:**
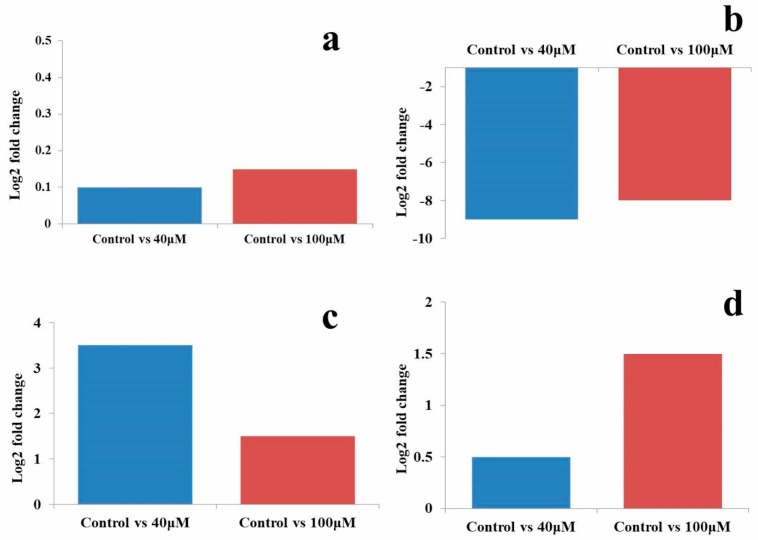
Log2 fold change obtained by NGS dataset in the untreated control vs. 40 and the untreated control vs. 100 μM EGCG treatments. (**a**) miR-21-5p; (**b**) hsa-miR-548o-5p; (**c**) hsa-miR-181c; (**d**) hsa-miR-212-5p microRNAs.

In microRNA sequencing data, an up-regulation of hsa-miR-181c by 3.51 and 1.5 log2 fold was observed in the untreated control vs. 40 and the untreated control vs. 100 μM EGCG treatments, respectively. Furthermore, a significant down-regulation of hsa-miR-548o-5p by 9 and 8 log2 fold change was noted in the untreated control vs. 40 and the untreated control vs. 100 μM EGCG treatments respectively (Figure 11). About 0.1 and 0.5 log2 fold change of hsa-miR-212-3p was observed in untreated control vs. 40 μM EGCG treatment. In hsa-miR-181c, the log2 fold change of 0.15 and 1.5 was noted in untreated control vs. 100 μM EGCG treatment. A comparative fold change between the qRT-PCR and sequencing dataset supports each other. Therefore, q-RT PCR results validate the present NGS dataset. 

**Figure 12:**
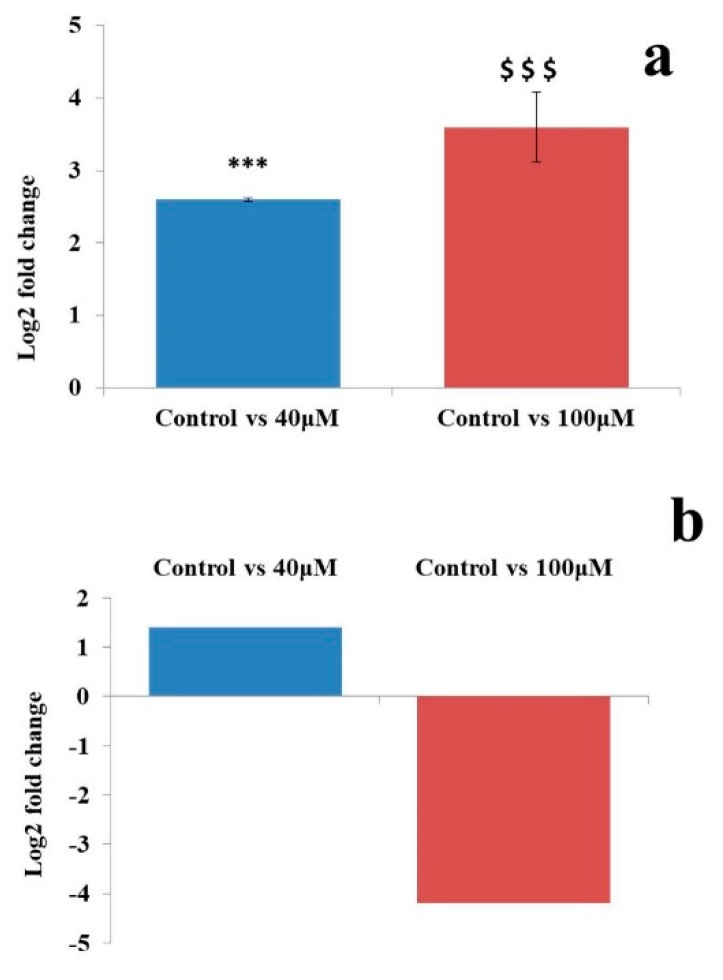
Validation of putative of novel microRNA TPP-A549-23 by qRT PCR. (**a**) Log2 fold change from qRT-PCR; (**b**) Log2 fold change from NGS dataset. Significant difference is indicated by ‘*’ between the untreated control and 40 µM EGCG treatment and ‘$’ between the untreated control and 100 µM EGCG treatment. Significance levels of *p* < 0.001 (‘***’; ‘$$$’) are denoted by repetition of these symbols.

A qRT-PCR analysis of the putative novel miRNA TPP-A549-23 showed 1.95 and 4.2 log2 fold change of expression in the untreated control vs. 40 and the untreated control vs. 100 μM EGCG treatments respectively (Figure 12). This fold change was compared with the log2 fold change obtained from the NGS data analysis. This sequence was up-regulated by 1.45 log2 fold change in the untreated control vs. 40 μM EGCG treatment and was down-regulated by 4.21-fold change in the untreated control vs. 100 μM EGCG treatment (Figure 12). 

It was observed that log2 fold change obtained in sequencing dataset was similar to the log2 fold change obtained from the qRT-PCR analysis. Furthermore, the present study also validates a novel microRNA sequence obtained in the NGS dataset. This qRT-PCR analysis attests to our computational analysis of NGS data.

### 2.8. KEGG and PANTHER Pathway Enrichment of Targets of Validated microRNAs 

**Figure 13:**
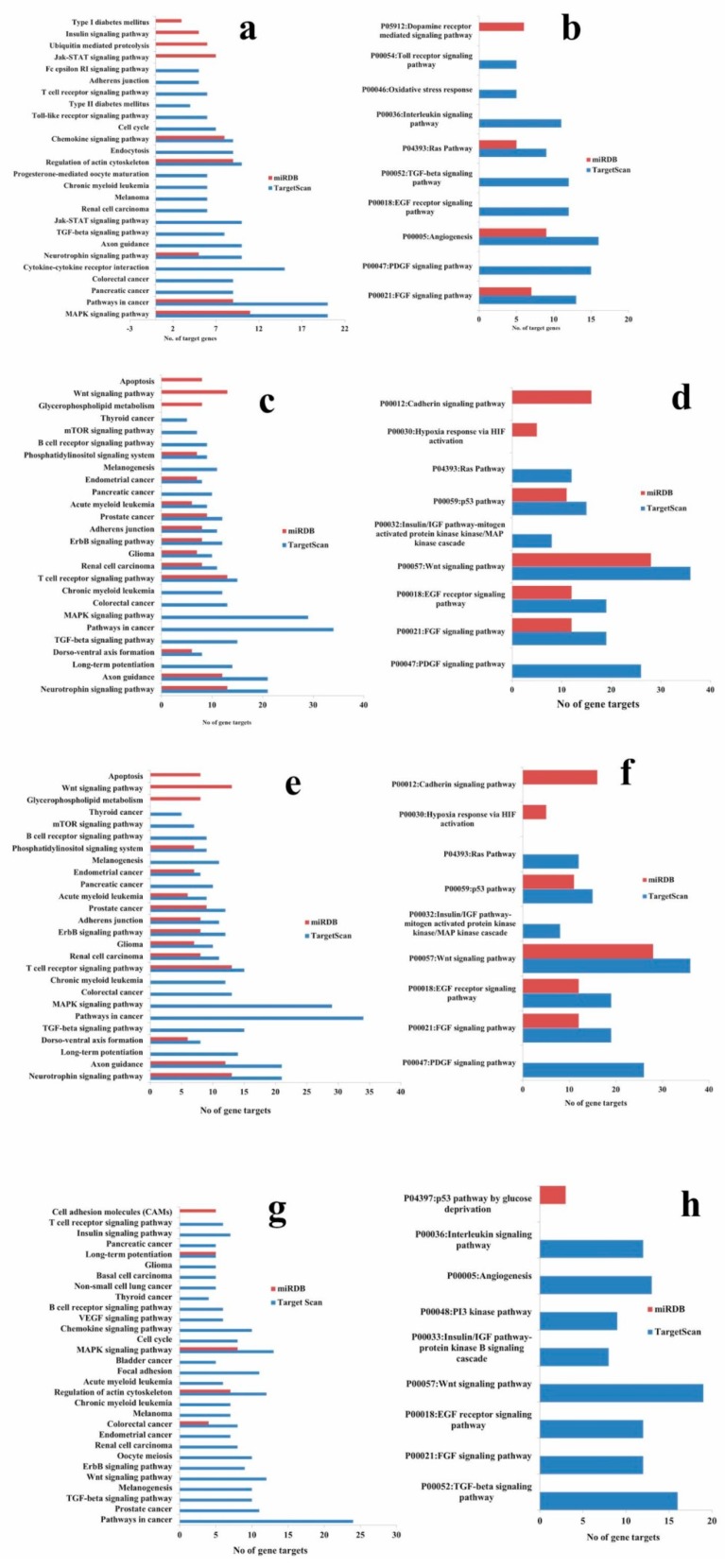
KEGG and PANTHER pathway enrichment of targets of validated microRNAs. (**a**) KEGG pathway analysis of hsa-miR-21-5p; (**b**) PANTHER pathway analysis of hsa-miR-21-5p; (**c**) KEGG pathway analysis of hsa-miR-548o-5p; (**d**) PANTHER pathway analysis of hsa-miR-548o-5p; (**e**) KEGG pathway analysis of hsa-miR-181c; (**f**) PANTHER pathway analysis of hsa-miR-181c; (**g**) KEGG pathway analysis of hsa-miR-212-5p; (**h**) PANTHER pathway analysis of hsa-miR-212-5p.

The high precision target prediction for hsa-miR-548o-5p, hsa-miR-181c, hsa-miR-212-5p, and hsa-miR-21-5p was carried out using TargetScan and miRDB target computational prediction software. Default cut off values were used for gene target prediction. KEGG and PANTHER pathway analysis were carried out using the Database for Annotation, Visualization and Integrated Discovery (DAVID) and the pathways were shortlisted. Pathway analysis for microRNAs was evaluated and common pathways predicted between TargetScan and miRDB are presented in Figure 13. Pathways in cancer, MAPK, regulation of actin cytoskeleton, wnt signaling, ErbB signaling, B-cell and T-cell receptor signaling, and long-term potentiation were the most significant pathways obtained in KEGG pathway analysis (Figure 13a,c,e,g). In addition, Ras signaling, angiogenesis, FGF signaling, wnt signaling, FGF, and PDGF (Platelet-derived growth factor) signaling pathway genes were reported in PANTHER pathway analysis (Figure 13b,d,f,h).

Furthermore, KEGG pathway analysis showed the MAPK signaling pathway as the target for hsa-miR-21-5p, hsa-miR-548o-5p, hsa-miR-181c, and hsa-miR-212-5p microRNAs (Table 4). Our analysis with PANTHER pathway did not show any common pathway among the microRNAs. Ras signaling pathway, wnt pathway, angiogenesis, p53, and EGF (Epidermal growth factor) receptor signaling pathway were the most significant pathways predicted by TargetScan and miRDB target list. 

MiRanda software was used for target prediction and pathway analysis of putative novel microRNA sequences. The common pathways found in the target prediction were wnt, angiogenesis, p-53, PI3K, and MAPK signaling pathways. The putative novel microRNA TPP-A549-23 targeted *FOPNL* (FGFR1OP N-Terminal Like), *ACVR1C* (Activin A Receptor Type 1C), and *CD38* (Cyclin-Dependent Kinase Inhibitor 2B) genes. Furthermore, the putative novel microRNA TPP-A549-24 targeted *RBM18* (RNA Binding Motif Protein 18) and *CDKN2B* (Cyclin-Dependent Kinase Inhibitor 2A). In addition, *APC*, *RADGEF2* (Rap Guanine Nucleotide Exchange Factor 2), *CNNM2* [Cyclin and CBS (cystathionine-beta-synthase) Domain Divalent Metal Cation Transport Mediator 2] *CORO2A* (Cronin 2A), *SEPT9* (Septin 9), *RAPGEF2* (Rap Guanine Nucleotide Exchange Factor 2), *JAK1* (Janus Kinase 1), and *WDR19* (WD Repeat Domain 19) were noted to be potential target genes for the putative novel microRNA TPP-A549-25. The pathway analysis showed that the putative microRNAs could play an important role in cell cycle proliferation, MAPK, Hedgehog, FOXO, and TGF-beta signaling cascade. We believe that these predicted putative novel microRNA sequences play a major role in cancer proliferation and metastasis. 

## 3. Discussion

To evaluate the changes of EGCG induced microRNA expression in A549 cells, an established in vitro model of human lung adenocarcinoma, and next-generation sequencing analysis were employed. The dose of EGCG used in the present study was decided based on cell cycle analysis and previous literature. EGCG is capable of causing G0/G1 phase arrest in many cancer cell lines including A549 [37,38,39,40]. The present cell cycle data analysis showed G0/G1 phase arrest at 40 μM EGCG treatment. Kweon et al. [41] reported that out of eight cell lines they studied, A549 showed no sign of apoptosis and was highly resistant even at 100 μM EGCG treatment. As much as 85% of the cell viability was sustained for 72 h at 40 μM EGCG treatment. We observed browning of the medium above 100 μM EGCG treatment. Previous data suggested that O_2_^−^ and quinones generation in the cell culture medium occurs due to the auto-oxidation properties of EGCG [42,43]. Hence, in the present study, we have chosen 40 μM and 100 μM EGCG concentrations. 

Nine hundred and fifty-nine microRNAs out of 1881 microRNAs (50.9%) reported in miRbase (as per Feb 2018) were expressed in A549 cells. Some microRNAs did not show differential expression while certain other microRNAs were significantly influenced by EGCG treatment. Therefore, EGCG dependent microRNA profiling was studied according to the three independent criteria namely log2 expression analysis, differentially up- and down-regulated microRNAs and putative gene targets of the microRNAs.

In the present study, the next-generation raw data was analyzed for greater than 2 log2 fold change of expression in known and putative novel microRNA sequences. In known microRNA dataset, out of 959 microRNAs, 115 (11.9%) and 134 (13.9%) microRNAs in 40  and 100 μM EGCG treatments respectively exhibited greater than 2 log2 fold change of expression (Table 2).In the putative novel microRNA dataset, out of 208 microRNAs, 4 (1.9%) and 3 (1.4%) microRNAs in 40  and 100 μM EGCG treatments respectively showed differential expression above 2 log2 fold change (Table 3). Interestingly, some microRNAs namely hsa-miR-125a-3p, hsa-miR-15b-3p, and hsa-miR-548av-3p in untreated control vs. 40 μM EGCG treatment and hsa-miR-125a-3p, hsa-miR-500a-3p, hsa-miR-7706, and hsa-miR-15b-3p in untreated control vs. 100 μM EGCG treatment exhibited greater than 6 log2 fold change of expression. These observations strongly support that EGCG can modulate multiple microRNAs. The putative novel microRNAs TPP-A549-14, TPP-A549-24, TPP-A549-27, and TPP-A549-28 in untreated control vs. 40 μM EGCG treatment and TPP-A549-32, TPP-A549-23, and TPP-A549-33 in untreated control vs. 100 μM EGCG treatment showed more than 2 log2 fold change of expression. 

Interestingly, the top 10 up-expressed microRNAs namely, hsa-miR-21-5p, hsa-let-7i-5p, hsa-miR-100-5p, hsa-miR-27b-3p, hsa-miR-151a-3p, hsa-miR-148a-3p, hsa-miR-30a-5p, hsa-miR-192-5p, hsa-miR-3529-3p, and hsa-miR-30d-5p were similar in untreated control and EGCG treatments. The hsa-miR-21 is the most commonly up-expressed microRNA in human cancers [44,45,46]. This microRNA is the first one to be named as “oncomir” [47] and was found to highly up-expressed in 540 clinical samples from cancer patients [48]. Yang et al. [49] reported significant up-regulation of hsa-miR-21 in NSCLC patients. In addition, they also reported that inhibition of hsa-miR-21 expression reduced cell proliferation, migration, and invasion in A549 cell. Up-expression of hsa-miR-21 in untreated control A549 cells further attests to our NGS data. Furthermore, due to treatment with EGCG, no significant down-regulation of hsa-miR-21 was observed in differential expression analysis of our NGS data (Figure 11a) which is validated by qRT-PCR analysis (Figure 10a). It is well known that hsa-miR-21 is an oncogene which plays an important role in programmed cell death and targeting apoptosis [50] but, in the present study no such apoptosis was observed on EGCG treatments. Therefore, we hypothesize that EGCG treatment has no modulatory effect on hsa-miR-21. Therefore, the present data support the non-apoptotic effect of EGCG in A549 cells. Another clinical study validated the chemoresistance role of hsa-mir-21 on platinum-based chemotherapy [51]. The persistent expression of hsa-miR-21 in untreated control and EGCG-treated A549 cells in the present study is supported by the clinical microarray data validated by Fujita et al. [51]. 

Let-7i, a member of the let-7 family, is an oncogenic driver of NSCLC. Increased expression of let-7i in the present study supports the statistically significant clinical study conducted on NSCLC [52]. Furthermore, a microarray analysis of primary lung cancer tumors and non-cancerous lung tissues revealed down-regulation of hsa-miR-181c [53]. In addition, Fujita et al. [51] reported the significance of hsa-miR-181c. It was observed that patients which did not respond to platinum-based chemotherapy had elevated hsa-miR-181c expression as compared to the well responsive patients. This study thus validated the chemoresistant role of hsa-miR-181c [51]. In the present study, EGCG treatment elevated the expression of hsa-miR-181c (Figure 9c). Moreover, this data was validated by qRT-PCR analysis (Figure 8c), where we found a significant up-regulation of hsa-miR-181c by 2.17 and 1.28 log2 fold change by 40 and 100 μM EGCG treatments, respectively. 

The direction of log2 fold change (up- or down-regulation) was analyzed in microRNA expression. About 53 and 69 microRNAs were up-regulated after 40 and 100 μM EGCG treatments, respectively. Hsa-miR-125a-3p was highly up-regulated with a log2 fold change of 7.12 and 7.47 in 40 and 100 μM EGCG treatments, respectively. Jiang et al. [54] showed an inverse relationship between hsa-miR-125a-3p with invasion and metastasis. Furthermore, it was marked that hsa-miR-125a-3p was down-regulated in NSCLC [54]. Significant up-regulation of hsa-miR-125a-3p was observed with EGCG treatment in A549 cells. As it was established that EGCG inhibits cell proliferation in A549 cells by causing G0/G1 phase arrest (Figure 1), up-regulation of signature microRNA hsa-miR-125a-3p attests to the role of EGCG in inhibiting cell proliferation and invasion.

Hsa-miR-548o-3p was significantly down-regulated by 9.12 and 8.12 log2 fold change in 40 and 100 μM EGCG treatments, respectively. Mir-548 family consist of 68 microRNAs and is the largest, and poorly conserved primate-specific gene family [55]. The reports on one of the members of mir-548 family, hsa-miR-548c-3p, revealed it as a functional biomarker in prostate and gastric cancer progression [54,56]. However, the exact function of hsa-miR-548o-3p in A549 cells is still unknown, but evidence supports the up-regulation of the mir-548 family in cancer progression. We noted down-regulation of hsa-miR-548o-3p by EGCG treatments (Figure 8b and Figure 9b) which indicated it as a potential biomarker for cancer progression. 

Hsa-miR-212 is a tumor suppressor microRNA which negatively regulates anti-apoptotic protein PED/PEA-15 [57]. A study by Jiang et al. [58] validated the biological role of hsa-miR-212/132 in A549 cells. Up-regulation of hsa-mir-212 blocked proliferation and migration and was observed to cause cell cycle arrest by modulating p21 and cyclin D1 expression [58]. An analysis of our NGS data supported the biological role of hsa-mir-212. We observed no significant expression of hsa-mir-212 in untreated control A549 cells. The lower expression of hsa-miR-212 signifies the cancerous property of A549 cells. In addition, negligible differential expression was observed in the EGCG-treated A549 cells. This indicated the fact that A549 cells are resistant to EGCG treatment. 

Notably, a significant modulation in hsa-miR-146b-3p was observed with EGCG treatment. Down-regulation of hsa-miR-146b-3p was observed with 40 μM EGCG treatment and a notable up-regulation was noticed in 100 μM EGCG treatment (Figure 6). A clinical study elucidated a down-regulation of hsa-miR-146b-3p in breast cancer tissues. Further findings revealed that hsa-miR-146b-3p’s over-expression suppressed migration, invasion, metastasis, and growth in breast cancer cell lines [59]. The principal findings on the role of hsa-miR-146-3p in untreated control A549 cells revealed the association of its lower expression with early-stage NSCLC recurrence [60]. Functional role of hsa-miR-146b-3p is still unexplored, but our data indicated its important role in tumor suppression. 

The goal of this study was to identify putative novel microRNA sequences in A549 cells and their potential differences in expression between untreated control and EGCG treatments. We reported, 4, 3, and 4 putative novel microRNA sequences in untreated control vs. 40, untreated control vs. 100, and 40 vs. 100 μM EGCG treatments respectively (Table 3). Only three putative microRNA sequences were persistently present between the untreated control vs. 40 and the untreated control vs. 100 μM EGCG treatments (Figure 9c). To validate the log2 fold change of the putative microRNA sequences, qRT-PCR analysis was done. The expression analysis of the putative novel microRNA TPP-A549-23 was done using qRT-PCR analysis. We observed similar log2 fold change between the untreated control vs. 40 and the untreated control vs. 100 μM EGCG treatments (Figure 8). The NGS dataset and validation confirmed the significant expression of this putative novel microRNA. Further validation of the sequence is required.

TargetScan and miRDB databases identified approximately 1200 potential target genes of microRNAs (data not shown). Next, we have used DAVID web-based gene ontology and pathway prediction software for further analysis. KEGG pathway and PANTHER pathway analysis were carried out with both the gene targets obtained by TargetScan and miRDB. 

Target prediction and pathway analysis of hsa-miR-21-5p, hsa-miR-548o-5p, hsa-miR-181c, and hsa-miR-212-5p revealed MAPK, pathways in cancer, Ras, FGF, wnt, and T-cell receptor signaling pathways as the most significant pathways in our study (Figure 13). Further analysis showed that these microRNAs commonly target the MAPK signaling pathway. The present study supports the functional role of hsa-miR-21-5p, hsa-miR-548o-5p, hsa-miR-181c, and hsa-miR-212-5p. Significant expression of these microRNAs in untreated control and EGCG-treated A549 cells demonstrated their important role in regulating cell proliferation.

For target prediction of putative novel microRNAs, we used miRanda 3.3a. Approximately 30 target genes were predicted for which pathway analysis was done. The putative novel microRNA sequences showed wnt, angiogenesis, p-53, PI3K, and MAPK signaling pathways as major targeting pathways. The common putative novel microRNAs differentially expressed between the control and EGCG treatments namely TPP-A549-23, TPP-A549-24, and TPP-A549-25 were shown to target cell cycle, MAPK, Hedgehog, FOXO, and TGF-beta signaling pathways. *FOPNL, ACVR1C, CD38. RBM18, CDKN2B, APC, RADGEF2, CNNM2, CORO2A, SEPT9, RAPGEF2, JAK1*, and *WDR19* genes were the potential targets for these putative novel microRNA sequences. These putative novel microRNA sequences showed an important role in cell cycle modulation and MAPK signaling cascade.

In summary, we demonstrated the important role of EGCG in modulating microRNA expression profiling. Substantial evidence from previous studies have provided an important role of EGCG in inhibiting cell proliferation in lung cancer, but its mechanistic insights on microRNA profiling remain incompletely understood. However, our results provided a useful approach for better understanding of EGCG induced microRNA modulation. Furthermore, exploration with transfection and human lung cancer tissue should be performed to validate the microRNA profiling and their predicted targets. 

## 4. Materials and Methods

### 4.1. Cell Culture and Treatment

Human non-small cell lung cancer A549 cells (purchased from National Centre for Cell Science, Pune, India) was cultured in F12 Ham-K medium containing 1X antibiotic-antimycotic solution and 10% FBS (Himedia, India) at 37 °C with 95% humidified atmosphere and 5% CO_2_. For cell cycle analysis, A549 cells were seeded in 60 mm cell culture dishes for 24 h in the F12-Ham-K medium. The cells were further treated with 20, 40, 80, 100, and 150 μM EGCG (Sigma Chemical Co. St. Louis, Missouri, United States, USA). After 24 h, cells were harvested and re-suspended in 100 μL PBS. The cells were fixed by drop-wise addition of 8–10 mL 75% ice-cold ethanol, continuous stirring, and incubating on ice for 15 min. After centrifugation, the cells were re-suspended in 200 μL PBS containing 0.04 mg/mL propidium iodide and 0.1 mg/mL RNase and was incubated for 30 min at 37 °C in dark. Cell cycle analysis was carried out on BD FACSVerse™ flow cytometer (ThermoFisher Scientific, Massachusetts, United States. 

### 4.2. Next-Generation Sequencing

A549 cells were treated with EGCG at the concentration of 40 and 100 μM. Untreated cells were used as a control. Cells were harvested for RNA extraction after 24 h of culture. Total RNA was extracted from the untreated and treated A549 cells using TRizol reagent (Takara Bio Inc., Kusatsu, Japan) following the manufacturer’s protocol. The small RNA library construction and deep sequencing were carried out at AgriGenome Labs Pvt. Ltd., Kochi, Kerala, India. NEBNext® Multiplex Small RNA Library Prep Set for Illumina was used for library construction. The raw counts and the normalized files were submitted in NCBI (National Center for Biotechnology Information) by accession number GSE110514. 

### 4.3. Classification and Differential Expression Analysis of MicroRNAs

The human genome (GRCh38) was used for the mapping of microRNA reads using Bowtie. Known miRNAs were identified using miRBase-21 database based on miRNA sequence similarity approach. The sequences were checked for other ncRNA contamination (rRNA, tRNA, snRNA, snoRNA, and piRNA). The novel microRNA prediction was evaluated by Mireap_0.22b [36]. Furthermore, secondary hairpin structures were predicted using mfold [61]. Expressed reads for each microRNAs were calculated and DESseq R software package was used for differential expression analysis. Differentially expressed microRNAs in control vs. 40, control vs. 100 and 40 vs. 100 μM EGCG treatments were determined by their expression in each sample [62]. The expressed reads in untreated control, 40, and 100 μM EGCG treatments were used to calculate the log2 fold change of expression between untreated control vs. 40, untreated control vs. 100, and 40 vs. 100 μM EGCG treatments.

### 4.4. Validation of MicroRNAs

To validate the expression of some of the significant known and putative novel microRNAs, total microRNA was isolated using miRNeasy Mini Kit (Qiagen Sciences, USA) from untreated control and EGCG-treated (40 and 100 μM) A549 cells. First-strand cDNA was synthesized using miScript PCR starter kit (Qiagen Sciences, Germantown, MD, USA) and SYBR green (nucleic acid strain) was used for qRT-PCR (ABI Prism 7000, ThermoFisher Scientific, Massachusetts, United States). The log2 fold change was calculated using the ΔΔCT method [63]. 

The comparative analysis of qRT-PCR and NGS was done to validate the microRNA expression profile obtained by NGS. The log2 fold change obtained by qRT-PCR of the known microRNAs namely miR-548o-3p, miR-212, miR-125a, and miR-181c was calculated and compared with the log2 fold change obtained in the NGS sequencing data. The log2 fold change of the putative novel microRNA TPP-A549-23 obtained by qRT-PCR and NGS was also compared for the sequence validation.

### 4.5. KEGG and PANTHER Pathway Enrichment of Targets of Validated MicroRNAs

The target prediction for known microRNAs was performed using TargetScan and miRDB [56] and the pathway analysis was done by The Database for Annotation, Visualization and Integrated Discovery (DAVID V 6.7). In addition, target prediction of the putative novel microRNA sequences was carried out using miRanda software [64]. 

### 4.6. Statistical Analysis

Statistical analysis was performed with one-way analysis of variance (ANOVA). The experimental data was represented as mean ± SD. The results were considered significant when *p* < 0.001, *p* < 0.01 or *p* < 0.05.

## 5. Conclusions

We used NGS to study a complete microRNA profiling of A549 cells and identified 958, 944 and 935 known microRNAs in the untreated control, 40, and 100 μM EGCG treatments, respectively. The oncogenic microRNAs were highly up-expressed in the untreated control and EGCG-treated A549 cells which are the part of broadly conserved let-7, hsa-miR-21, and hsa-miR-30 seed families. The up-expressions of these oncogene microRNAs with EGCG treatment indicated resistance character of A549 cells for EGCG. The differential expression analysis of microRNAs identified highly up-regulated hsa-miR-125a-3p and highly down-regulated hsa-miR-548o-3p in the untreated control vs. 40 and the untreated control vs. 100 μM EGCG treatments, respectively. A similar log2 fold change in the comparative analysis between NGS and qRT-PCR of randomly selected known and putative novel microRNAs validated the NGS data. Our data indicated EGCG as an effective natural compound which regulates microRNA profile in A549 cells. This study also attested the modulation of microRNAs by EGCG which regulates cell cycle and inhibits cell proliferation and metastasis. KEGG and PANTHER pathway analysis revealed the MAPK pathway as the most potent targeted pathway by EGCG modulated microRNAs. The findings explored an important role of EGCG in microRNA regulation, which targets MAPK cascade. Furthermore, the putative novel microRNA sequences reported in this study can be a novel approach towards the microRNA targeting gene therapies. 

## Figures and Tables

**Table 1:** Top 10 putative microRNA sequences expressed in the untreated control, 40, and 100 μM EGCG treatments.

Putative Novel MicroRNA ID	Sequences	Chromosome Number	Precursor Start	Precursor End	Mature Start	Mature End	Precursor Sequence	MFE (Kcal/mol)
**A549 Control**								
TPP-A549-1	CCAGGAUGCACGCUCGCUGGGCU	17	375,2068	375,2154	375,2078	375,2100	CCGUUGCCACCCAGGAUGCACGCUCGCUGGGCUGGUGGGUGCCCACUCCGUUGCCACCCAGGAUGCACGCUCACUGGCCAGGGACCU	−30.7
TPP−A549-2	CGCUUCGUGUAGACCCUCCAC	15	748,733,11	748,733,96	748,733,21	748,733,41	GACCCAGCGGCGCUUCGUGUAGACCCUCCACUUCCGGGAGCGAGGCAGCGGUUCUGGCGCAGGCGCGAUGCCCUCCCCCGAGGGCG	−35.4
TPP-A549-3	CUUAGAAUUCCGAUGCUGGGG	22	267,618,39	267,619,28	267,618,98	267,619,18	AGAUUCUGGACUUAGAAUUCCGAUGCUGGGGCCAGGCACAGUGGCUCACACCUGUAAUCCCAGUACUUUGGGAAGCCGAGGCGGGAGGAU	−28.8
TPP-A549-4	AGGCUGUAAGGCCACACGGGGUG	18	374,244,03	374,244,95	374,244,63	374,244,85	CCUCCAAGGCAGGCUGUAAGGCCACACGGGGUGGAAGGCCAGAGCUCCUUCUCCUCCUUCUGCUCUCUGGGUAUUGCCACCAAGCCCUGUACC	−32.8
TPP-A549-5	CCUAGGACCAUCACAGGCUGA	16	888,546,38	888,547,12	888,546,82	888,547,02	AACCUCCCACUGGGUCUGCUGACUGCAGGGGUGACUUGGAGUCACCUAGGACCAUCACAGGCUGAGUGAGGAUGU	−34.3
TPP-A549-6	AUACUAUCUAGCUUUGGGUAGUA	6	705,117,52	705,118,32	705,117,62	705,117,84	GCGUAGGAGUAACAGGCUGUAUUAUCUAGUUUAGGUGUGUAGCAGGCUAUACUAUCUAGCUUUGGGUAGUAUAUUCUAUGA	−25.3
TPP-A549-7	CAGAUGGUUGCUGAUCUGUGCA	4	107,393,112	107,393,209	107,393,122	107,393,143	CAGGGUGUCCCAGAUGUCUCUGCAGCUUCCUCCUUGCAUCAGAUGCCAGCAUCCAGUGACUCGAUCCAGAUGGUUGCUGAUCUGUGCAGCAGAUCCUC	−29
TPP-A549-8	AGUGUUUAAGAUCCAAGUGUUG	4	911,143,10	911,143,99	911,143,68	911,143,89	UGUGCUUCUAAGUGUUUAAGAUCCAAGUGUUGAUAAUGGAAGUCACAUUAUCAACAUAAUUGGUAUCUAUAGAUGCUCUUUGGAUUCCAA	−29.2
TPP-A549-9	UUGGUGGUGUACACGGAGCAG	19	385,161,25	385,162,07	385,161,77	385,161,97	CCGCGGAGACUUGGUGGUGUACACGGAGCAGGCGUUGUACUCGUUCAGCUGCGGCUCCAGGAACGCCACCGGCAUGGCUGCUG	−36.6
TPP-A549-10	CCAGGACUCGAACCCUCCACCC	8	144,458,110	144,458,200	144,458,169	144,458,190	ACAUGGGUCUGAGAGGGACUGGCACGUUGUCCAAGGCCGCACAGCUGUUAAGCAUGAAGCCAGGACUCGAACCCUCCACCCAGGCUGGAUU	−29.8
**40 μM EGCG Treatment**								
TPP-A549-11	CUAAGACUGCAGUGAGCCUUGGU	16	589,8984	589,9051	589,8994	589,9016	GCCCAGGAGACUAAGACUGCAGUGAGCCUUGGUUUGCAGUGAGCCUCACUGCUUUUAGUUUCAGAGAU	−25.1
TPP-A549-12	CACCUGUAGUUCCAUCUACUCCAG	7	789,447,92	789,44892	789,448,59	789,448,82	UAGUGGCGUGCACCUGUAGUUCCAUCUACUCCAGAGGCUGAGGUGGGGGGAUCGUUUGAGCUUGGGAGAUGGAGGUUGCAGUGAGCAGUGAUUGUGCCACU	−38.51
TPP-A549-13	CAAGCCAUUGAUCCCAAAGUCA	1	535,110,24	535,111,16	535,110,85	535,111,06	CCAGAGCCCCCAAGCCAUUGAUCCCAAAGUCAUUUUAGCCCCUGUGAACGGGGCCCAGGGGCUUAGGAAGGUAGCAUGAGAAGGGAACCUGGC	−29.3
TPP-A549-14	CUCUGAACUGGGUAGAUCUGUC	2	181,190,09	181,190,98	181,190,19	181,190,40	AACAGGGUAGCUCUGAACUGGGUAGAUCUGUCUGUCAAGAGGAACGCUCUAGAAAGCUCAGGACUCUCCUGCACAGGGCUUAGCCCACUG	−31.6
TPP-A549-9	UUGGUGGUGUACACGGAGCAGGC	19	385,161,25	385,162,07	3851,6175	385,161,97	CCGCGGAGACUUGGUGGUGUACACGGAGCAGGCGUUGUACUCGUUCAGCUGCGGCUCCAGGAACGCCACCGGCAUGGCUGCUG	−36.6
TPP-A549-8	AGUGUUUAAGAUCCAAGUGUUG	4	911,143,10	911,143,99	911,143,68	911,143,89	UGUGCUUCUAAGUGUUUAAGAUCCAAGUGUUGAUAAUGGAAGUCACAUUAUCAACAUAAUUGGUAUCUAUAGAUGCUCUUUGGAUUCCAA	−29.2
TPP-A549-7	CAGAUGGUUGCUGAUCUGUGCA	4	107,393,112	107,393,209	107,393,122	107,393,143	CAGGGUGUCCCAGAUGUCUCUGCAGCUUCCUCCUUGCAUCAGAUGCCAGCAUCCAGUGACUCGAUCCAGAUGGUUGCUGAUCUGUGCAGCAGAUCCUC	−29
TPP-A549-3	CUAGGACCAUCACAGGCUGA	16	888,546,38	888,547,12	888,546,83	888,547,02	AACCUCCCACUGGGUCUGCUGACUGCAGGGGUGACUUGGAGUCACCUAGGACCAUCACAGGCUGAGUGAGGAUGU	−34.3
TPP-A549-15	AAUUUCUGAUCCUGGCCAUAUUC	1	414,2086	414,2164	414,2096	414,2118	ACAGAGGAUAGAGUUGGGCAGCAUCAGAAAGCCCAGCUUCCUCUGGAAUUUCUGAUCCUGGCCAUAUUCACUUCCUGUG	−34.5
TPP-A549-16	GCUAAAACUGGUCAAAGUGCUG	6	117,578,916	117,579,011	117,578,926	117,578,947	UGUUUUGGAGAAAGAAGUACAUGAUCAGCUUUUACAGCUGCACUCUAUUCAGCUGCAGCUUCAUGCUAAAACUGGUCAAAGUGCUGACUCUGGUAC	−28.5
**100 μM EGCG Treatment**								
TPP-A549-17	AUCAGUGGUUCUCAAUGUUCUUUU	1	797,2080	797,2153	797,2120	797,2143	GUUAUGGGCCAGAGGUCCUGGGGACUUGAUACAUUCGAGUAUCAGUGGUUCUCAAUGUUCUUUUGGUGCACUUG	−27.5
TPP-A549-11	CUAAGACUGCAGUGAGCCUUGGU	16	589,8984	589,9051	589,8994	589,9016	GCCCAGGAGACUAAGACUGCAGUGAGCCUUGGUUUGCAGUGAGCCUCACUGCUUUUAGUUUCAGAGAU	−25.1
TPP-A549-18	CAUCCACCUGGUCCAGCUCGC	16	317,07819	317,079,08	317,078,29	317,078,49	ACCUGGGAGGGACCAAGGCUGGGAGGGUGAGAGGCCAUGCCCACAAAAGCCUCAGGCUACAUCCACCUGGUCCAGCUCGCCAAGAUGGGA	−34.2
TPP-A549-19	GCUGUUGUUGCUGUGGUUGUGGCU	2	182,3300	182,3382	182,3310	182,3333	UGGCUGUGAGGCUGUUGUUGCUGUGGUUGUGGCUGCAACCUUCAUUGUCAUAGAUAUAGCUGUGGCAGCUGUAGUUGUAGCCU	−34.02
TPP-A549-7	CAGAUGGUUGCUGAUCUGUGCA	4	107,3931,12	107,393,209	107,393,122	107,393,143	CAGGGUGUCCCAGAUGUCUCUGCAGCUUCCUCCUUGCAUCAGAUGCCAGCAUCCAGUGACUCGAUCCAGAUGGUUGCUGAUCUGUGCAGCAGAUCCUC	−29
TPP-A549-20	GCUUAGCUAAGAAUACUUAUAAUU	3	997,767,29	997,768,24	997,767,39	997,767,62	AGCUUGGGCUGCUUAGCUAAGAAUACUUAUAAUUGUUUCUUGAUUAUAUGCUAAACAAGGGGUGGAUUCAUGAGUUUUCUGGGAAAGGGGUAGGCA	−25.5
TPP-A549-21	GAAGCUGUUGGACUAGAAAAAAUU	4	106,688,766	106,688,864	1066,888,31	106,688,854	UACCCUCUGAUAGGUUUUCUCAGCUGCAGCUUCACUGUGUGCAUAACCUCACUGUGGGGCUCAAGGAAGCUGUUGGACUAGAAAAAAUUUCAUUGCUGC	−25.6
TPP-A549-1	CCAGGAUGCACGCUCGCUGGGC	17	375,2068	375,2154	375,2078	375,2099	CCGUUGCCACCCAGGAUGCACGCUCGCUGGGCUGGUGGGUGCCCACUCCGUUGCCACCCAGGAUGCACGCUCACUGGCCAGGGACCU	−30.7
TPP-A549-22	GUAAAGACGUUGAUGCUGCUA	17	857,0265	857,0362	857,0332	857,0352	AGGAGUUUCAUAUACUAUCUCUAUCAUAUAGACAGGGCAAUGUCUUCACCUCCACUUUCAGAGGAUGGUAAAGACGUUGAUGCUGCUAACUUGCUCAG	−26.22
TPP-A549-26	UCUUCAUCUGGUUUGUGAACUUUU	17	569,836,22	569,837,13	569,836,32	569,836,55	GCGGUUAACAUCUUCAUCUGGUUUGUGAACUUUUUACUGCUUAGGAAAUCUUAAGAUACAAAGGGCAUAUGAUCAGGGGGAUGUAUAGCCUU	−31.7

**Table 2:** List of known microRNAs with greater than 2 log2 fold change expression after 40 and 100 μM EGCG treatments.

Treatments Compared	No. of MicroRNAs	MicroRNAs	
		Up-Regulated	Down-Regulated
Control vs. 40 μM	115	hsa-miR-125a-3p, hsa-miR-15b-3p, hsa-miR-548av-3p, hsa-miR-1294, hsa-miR-335-5p, hsa-miR-937-3p, hsa-miR-3118, hsa-miR-125a-5p, hsa-miR-769-3p, hsa-miR-2277-5p, hsa-miR-30c-2-3p, hsa-miR-24-1-5p, hsa-miR-181a-2-3p, hsa-miR-105-5p, hsa-miR-181c-5p, hsa-miR-365a-3p, hsa-miR-374c-5p, hsa-miR-3922-5p, hsa-miR-26b-5p, hsa-miR-449c-5p, hsa-miR-556-3p, hsa-miR-758-3p, hsa-miR-151a-5p, hsa-miR-331-5p, hsa-miR-942-5p, hsa-miR-20b-5p, hsa-miR-548a-3p, hsa-miR-103a-2-5p, hsa-miR-323b-3p, hsa-miR-421, hsa-miR-183-3p, hsa-miR-940, hsa-miR-935, hsa-miR-9-5p, hsa-let-7f-1-3p, hsa-miR-1468-5p, hsa-miR-4510, hsa-miR-5009-5p, hsa-miR-589-3p, hsa-miR-6747-3p, hsa-miR-195-5p, hsa-miR-486-3p, hsa-miR-454-3p, hsa-miR-3173-5p, hsa-miR-543, hsa-miR-125b-2-3p, hsa-miR-155-5p, hsa-miR-3179, hsa-miR-1976, hsa-miR-598-3p, hsa-miR-548al, hsa-miR-6853-3p, hsa-miR-6733-5p	hsa-miR-548o-3p, hsa-miR-96-5p, hsa-miR-185-3p, hsa-miR-1299 hsa-miR-22-5p, hsa-miR-195-3p, hsa-miR-653-5p, hsa-miR-425-3p, hsa-miR-215-5p, hsa-miR-7-1-3p, hsa-miR-132-5p, hsa-miR-424-5p, hsa-miR-3613-5p, hsa-miR-30b-5p, hsa-miR-548f-5p, hsa-miR-26a-2-3p, hsa-miR-18a-3p, hsa-miR-30b-3p, hsa-miR-654-3p, hsa-miR-140-5p, hsa-miR-6720-3p, hsa-miR-493-5p, hsa-miR-122-5p, hsa-miR-548n, hsa-miR-580-3p, hsa-miR-6723-5p, hsa-miR-1303, hsa-miR-597-3p, hsa-miR-1293, hsa-miR-10a-3p, hsa-miR-4473, hsa-miR-3163, hsa-miR-3191-5p, hsa-miR-365a-5p, hsa-miR-100-3p, hsa-miR-191-5p, hsa-miR-130b-3p, hsa-miR-20a-3p, hsa-miR-3134, hsa-miR-23a-5p, hsa-miR-556-5p, hsa-miR-4488, hsa-miR-491-5p, hsa-miR-378i, hsa-miR-3942-5p, hsa-miR-548j-3p, hsa-miR-6806-3p, hsa-miR-200a-3p, hsa-miR-212-3p, hsa-miR-105-3p, hsa-miR-1273g-3p, hsa-miR-34a-3p, hsa-miR-342-3p, hsa-miR-192-3p, hsa-miR-29c-5p, hsa-let-7d-5p, hsa-miR-1305, hsa-miR-146b-3p, hsa-miR-578, hsa-miR-6811-5p, hsa-miR-552-3p, hsa-miR-4755-3p, hsa-miR-3127-5p
40 Control vs. 100 μM	121	hsa-miR-548ah-3p, hsa-miR-96-5p, hsa-miR-500a-3p, hsa-miR-185-3p, hsa-miR-22-5p, hsa-miR-7706, hsa-miR-30e-3p, hsa-miR-532-5p, hsa-miR-502-3p, hsa-miR-1299, hsa-miR-4677-3p, hsa-miR-582-5p, hsa-miR-195-3p, hsa-miR-424-5p, hsa-miR-425-3p, hsa-miR-3613-5p, hsa-miR-653-5p, hsa-miR-424-3p, hsa-miR-152-5p, hsa-miR-32-3p, hsa-miR-18a-3p, hsa-miR-548n, hsa-miR-493-5p, hsa-miR-30b-3p, hsa-miR-618, hsa-miR-654-3p, hsa-miR-548f-5p, hsa-miR-140-5p, hsa-miR-92a-1-5p, hsa-miR-146b-3p, hsa-miR-548g-3p, hsa-miR-26a-2-3p, hsa-miR-10a-3p, hsa-miR-598-3p, hsa-miR-4502, hsa-miR-423-3p, hsa-miR-191-5p, hsa-miR-6720-3p, hsa-miR-3664-3p, hsa-miR-130b-5p, hsa-miR-3150b-3p, hsa-miR-4473, hsa-miR-2682-3p, hsa-miR-365a-5p, hsa-miR-215-5p, hsa-miR-4742-3p, hsa-miR-6511a-3p, hsa-miR-28-5p, hsa-miR-580-3p, hsa-miR-3942-5p, hsa-miR-6775-3p, hsa-miR-6818-3p, hsa-miR-3591-5p, hsa-miR-625-3p, hsa-miR-6868-3p, hsa-miR-597-3p, hsa-miR-381-3p, hsa-miR-127-3p, hsa-miR-1229-3p, hsa-miR-200a-3p, hsa-miR-23a-5p, hsa-miR-3120-3p, hsa-miR-433-3p, hsa-miR-4677-5p, hsa-miR-655-3p, hsa-miR-5091, hsa-miR-627-3p, hsa-miR-548aj-5p	hsa-miR-3184-5p, hsa-miR-548av-3p, hsa-miR-27a-3p, hsa-let-7d-3p, hsa-miR-548f-3p, hsa-miR-361-5p, hsa-miR-365a-3p, hsa-miR-3607-3p, hsa-miR-584-5p, hsa-miR-3922-5p, hsa-miR-148b-5p, hsa-miR-7641, hsa-miR-181b-3p, hsa-miR-3158-3p, hsa-miR-105-5p, hsa-miR-4796-5p, hsa-miR-1-3p, hsa-miR-151a-5p, hsa-miR-374a-5p, hsa-miR-1293, hsa-miR-4775, hsa-miR-574-3p, hsa-miR-331-5p, hsa-miR-107, hsa-miR-4717-5p, hsa-miR-363-3p, hsa-let-7f-2-3p, hsa-miR-454-5p, hsa-miR-103a-2-5p, hsa-miR-374c-5p, hsa-miR-6783-3p, hsa-miR-1185-2-3p, hsa-miR-935, hsa-miR-1468-5p, hsa-miR-744-3p, hsa-miR-3131, hsa-miR-4762-3p, hsa-miR-873-5p, hsa-miR-3923, hsa-let-7a-2-3p, hsa-miR-769-3p, hsa-miR-1249-5p, hsa-miR-142-5p, hsa-miR-181c-5p, hsa-miR-3179, hsa-miR-33a-5p, hsa-miR-3688-3p, hsa-miR-3944-3p, hsa-miR-410-3p, hsa-miR-4684-5p, hsa-miR-548ay-3p, hsa-miR-636, hsa-miR-653-3p, hsa-miR-6843-3p
Control vs. 100 μM	134	hsa-miR-125a-3p, hsa-miR-500a-3p, hsa-miR-7706, hsa-miR-15b-3p, hsa-miR-598-3p, hsa-miR-532-5p, hsa-miR-502-3p, hsa-miR-30e-3p, hsa-miR-181a-2-3p, hsa-miR-1294, hsa-miR-24-1-5p, hsa-miR-424-3p, hsa-miR-335-5p, hsa-miR-32-3p, hsa-miR-125a-5p, hsa-miR-618, hsa-miR-3664-3p, hsa-miR-3118, hsa-miR-582-5p, hsa-miR-4677-3p, hsa-miR-2277-5p, hsa-miR-30c-2-3p, hsa-miR-152-5p, hsa-miR-92a-1-5p, hsa-miR-421, hsa-miR-937-3p, hsa-miR-423-3p, hsa-miR-26b-5p, hsa-miR-548g-3p, hsa-miR-130b-5p, hsa-miR-625-3p, hsa-miR-9-5p, hsa-miR-556-3p, hsa-miR-758-3p, hsa-miR-769-3p, hsa-miR-5009-5p hsa-miR-2682-3p, hsa-miR-454-3p, hsa-miR-4502, hsa-miR-222-3p, hsa-miR-26a-1-3p, hsa-miR-3140-3p, hsa-miR-3591-5p, hsa-miR-548al, hsa-miR-940, hsa-miR-1255a, hsa-let-7f-1-3p, hsa-miR-146b-3p, hsa-miR-6868-3p, hsa-miR-548x-3p, hsa-miR-1285-5p, hsa-miR-1304-3p, hsa-miR-2116-3p, hsa-miR-28-5p, hsa-miR-543, hsa-miR-1229-3p, hsa-miR-1290, hsa-miR-155-5p, hsa-miR-181b-2-3p, hsa-miR-1976 hsa-miR-3120-3p, hsa-miR-3129-5p, hsa-miR-3174, hsa-miR-323b-3p, hsa-miR-432-5p, hsa-miR-548a-3p, hsa-miR-589-3p, hsa-miR-655-3p hsa-miR-6747-3p	hsa-miR-548o-3p, hsa-miR-3184-5p, hsa-miR-27a-3p, hsa-miR-548f-3p, hsa-let-7d-3p, hsa-miR-1293, hsa-miR-7641, hsa-miR-3607-3p, hsa-miR-132-5p, hsa-miR-7-1-3p, hsa-miR-3158-3p, hsa-miR-361-5p, hsa-miR-6723-5p, hsa-miR-1-3p, hsa-miR-148b-5p, hsa-miR-30b-5p, hsa-miR-584-5p, hsa-miR-491-5p, hsa-miR-181b-3p, hsa-miR-574-3p, hsa-miR-1303, hsa-let-7a-2-3p, hsa-miR-215-5p, hsa-miR-548q, hsa-miR-629-3p, hsa-miR-1262, hsa-miR-107, hsa-miR-374a-5p, hsa-let-7d-5p, hsa-miR-3688-3p, hsa-miR-4793-3p, hsa-miR-744-3p, hsa-miR-4796-5p, hsa-miR-20a-3p, hsa-miR-122-5p, hsa-miR-6761-5p, hsa-miR-6806-3p, hsa-miR-135b-5p, hsa-miR-4775, hsa-miR-450a-1-3p, hsa-miR-142-5p, hsa-miR-378i, hsa-miR-7705, hsa-miR-548ay-3p, hsa-miR-548j-3p, hsa-miR-1273g-3p, hsa-miR-130b-3p, hsa-miR-4488, hsa-miR-345-5p, hsa-let-7f-2-3p, hsa-miR-192-3p, hsa-miR-1249-5p, hsa-miR-136-5p, hsa-miR-184, hsa-miR-3127-5p, hsa-miR-3163, hsa-miR-3191-5p, hsa-miR-3654,hsa-miR-3691-5p, hsa-miR-3944-3p, hsa-miR-4477b, hsa-miR-4787-3p, hsa-miR-485-5p, hsa-miR-578, hsa-miR-6737-3p, hsa-miR-6843-3p

**Table 3:** Putative novel microRNA sequences with greater than 2 log2 fold change in expression after 40 and 100 μM EGCG treatments.

Treatment Compared	No. of MicroRNAs	Putative Novel Micro RNA ID	Log2 Fold Change	microRNA Sequences		Chromosome Number	Precursor Sequence Start	Precursor Sequence End	Mature Sequence Start	Mature Sequence End	Precursor Sequence	MFE (Kcal/mol)
				Up-Regulated	Down-Regulated							
Control vs. 40 μM	4	TPP-A549-26	2.52	CUCUGAACUGGGUAGAUCUGUC		2	181,190,09	181,190,98	181,190,19	181,190,40	AACAGGGTAGCTCTGAACTGGGTAGATCTGTCTGTCAAGAGGAACGCTCTAGAAAGCTCAGGACTCTCCTGCACAGGGCTTAGCCCACTG	−31.6
		TPP-A549-24	-2.5		AAUAGGAAUUGAGAGAAGUG	4	107,162,755	107,162,845	107,162,765	107,162,784	CTCATGTTTTACTTCCTTTTCCTATTTTGTTACACTAGCTAGGGCTTCTAGTAGAGTAATGAATAGGAATTGAGAGAAGTGATATTTTGGC	−27.5
		TPP-A549-27	-3.34		CUUAGAAUUCCGAUGCUGGGG	22	267,618,39	267,619,28	267,618,98	267,619,18	AGATTCTGGACTTAGAATTCCGATGCTGGGGCCAGGCACAGTGGCTCACACCTGTAATCCCAGTACTTTGGGAAGCCGAGGCGGGAGGAT	−28.8
		TPP-A549-28	-2.23		GAAAAACCUGAGCUGAUGGAC	5	140,056,955	140,057,037	140,057,007	140,057,027	TCTAGATGATGAAAAACCTGAGCTGATGGACAATGCCCGTAAGTGTATTGTGATATTGCAGCTCAGGTTTTTCATTAATGTAA	−36.3
40 vs. 100 μM	4	TPP-A549-29	2.2	GAGGUAGAGCAAGGGCCCCU		13	394,145,14	394,146,06	394,145,24	394,145,43	GGCTGGAGTTGAGGTAGAGCAAGGGCCCCTGCATTTGGTGTTATCAGTCTTCTGGGACTTTCTTGGGGTACTGTTTTCACTTCTTCTTCTTAT	−27.2
		TPP-A549-30	2.6	UUCAGGUUGAAGCUCUUGCACA		14	102,442,311	102,442,392	102,442,321	102,442,342	GGGATGAGTCTTCAGGTTGAAGCTCTTGCACAGCTGGCTCTCTCCTAGCTGTGTAAGAACCTCTGGCCTGGGTGCACAGCCA	−38
		TPP-A549-31	−2.32		CCAGCCUACUGGAGGAUAAGAGGA	8	102,442,311	102,4423,92	102,442,321	102,442,342	GGGATGAGTCTTCAGGTTGAAGCTCTTGCACAGCTGGCTCTCTCCTAGCTGTGTAAGAACCTCTGGCCTGGGTGCACAGCCA	−38
		TPP-A549-32	−2.22		CUAGGACCAUCACAGGCUGA	16	983,936,55	983,937,34	983,937,01	983,937,24	CTCTGGTTCCCCAGCCTACTGGAGGATAAGAGGATATAAAGGTCTCTTATCCTCCAGTAGACTAGGGAGCCAGAGCTGGT	−53.7
Control vs. 100 μM	3	TPP-A549-33	2.43	GAGGUAGAGCAAGGGCCCCU		13	394,145,14	394,146,06	394,145,24	394,145,43	GGCTGGAGTTGAGGTAGAGCAAGGGCCCCTGCATTTGGTGTTATCAGTCTTCTGGGACTTTCTTGGGGTACTGTTTTCACTTCTTCTTCTTAT	−27.2
		TPP-A549-23	−4.2		CUUGCAAUUACUUUUGCACCAACU	4	158,492,16	158,493,08	158,492,26	158,492,49	TAAATTATTAGGTTGGTTCAAAAATAATTGTGGTTTTGCCATTCCTTTCGGTGGCAAAACTTGCAATTACTTTTGCACCAACTTAAATATATA	−37.4
		TPP-A549-34	−4.2		UAGGUGGAUUUGUGUGAUGAGCU	9	195,7951	195,8044	195,7961	195,7983	AGGCCTGCCATAGGTGGATTTGTGTGATGAGCTAAGGAGTTTCCCAAGCAGATATCATCCTAATGGAAAAGAGAACCAAGCACAAGCTCGGTCA	−25.5

**Table 4:** List of common pathways in TargetScan and mirDB.

MicroRNAs	KEGG Pathways Common in TargetScan and miRDB	PANTHER Pathways Common in TargetScan and miRDB	KEGG Pathway Common in all the MicroRNAs	PANTHER Pathways Common in all the microRNAs
Hsa-miR-21-5p	MAPK signaling pathway, Regulation of actin cytoskeleton, Chemokine signaling pathway, Pathways in cancer, Neurotrophin signaling pathway	P00021: FGF signaling pathway, P00005: Angiogenesis, P04393: Ras Pathway	MAPK signaling pathway	Nil
Hsa-miR-548o-5p	Small cell lung cancer, Colorectal cancer, Pancreatic cancer, Renal cell carcinoma, T cell receptor signaling pathway, the Hedgehog signaling pathway, Long-term potentiation, TGF-beta signaling pathway, ErbB signaling pathway, Endometrial cancer, B cell receptor signaling pathway, Acute myeloid leukemia, Glioma, Non-small cell lung cancer, MAPK signaling pathway, Axon guidance, Apoptosis, Wnt signaling pathway	P00057: Wnt signaling pathway		
Hsa-miR-181c	Neurotrophin signaling pathway, Axon guidance, Long-term potentiation, Dorso-ventral axis formation, TGF-beta signaling pathway, Pathways in cancer, MAPK signaling, pathway Colorectal cancer, Chronic myeloid leukemia, T cell receptor signaling pathway, Renal cell carcinoma, Glioma, ErbB signaling pathway, Adherens junction, Prostate cancer, Acute myeloid leukemia, Pancreatic cancer, Endometrial cancer, Phosphatidylinositol signaling system	P00021: FGF signaling pathway, P00018: EGF receptor signaling pathway, P00057: Wnt signaling pathway P00032: Insulin/IGF pathway-mitogen activated protein kinase/MAP kinase cascade, P00059: p53 pathway		
Hsa-miR-212-5p	Colorectal cancer, Regulation of actin cytoskeleton, MAPK signaling pathway, Long-term potentiation	Nil		
